# Mature cystic teratoma without intratumoral fat: A diagnostic dilemma

**DOI:** 10.1016/j.radcr.2023.06.005

**Published:** 2023-06-23

**Authors:** Sandra C M, Breman Anil Peethambar

**Affiliations:** a Department of Radiology, MES Medical College, Palachode Post, Perinthalmanna, Kerala, India; b Madras Medical College and Government General Hospital, Emergency Department, Chennai, Tamil Nadu, India

**Keywords:** Teratoma, CT, Torsion, Intratumoral fat, Dermoid

## Abstract

Teratomas are the most common benign ovarian neoplasms in young women. Typical computed tomography imaging findings include fat, fat fluid level, tooth or calcification, rokitansky nodule, floating balls sign, and tufts of hair. They can have unusual imaging features leading to diagnostic dilemmas. Studies have shown the presence of intratumoral fat to be specific to ovarian cystic teratoma. However, there are reports in the literature of mature cystic teratoma that do not contain fat in the lumen of the cyst which can hinder an accurate diagnosis. They can be associated with various complications like torsion, rupture, malignant transformation, infection, and autoimmune hemolytic anemias. Presented here is a case of mature cystic teratoma without visible intracystic fat which underwent torsion.

## Introduction

Mature cystic teratoma (MCT) is a common neoplasm of the ovary in young women with a wide variety of presentations ranging from pure cystic mass to complex solid cystic mass. The detection of intratumoral fat is the key diagnostic imaging feature. Frequently associated complications include torsion and malignant transformation [1]. We report a case of MCT without fat in the intracystic component which presented with torsion.

## Case report

A 57-year-old previously healthy woman was admitted with complaints of lower abdominal pain since the previous day. She had a single episode of vomiting. There was no history of fever, bleeding per vaginum, loose stools, or food intake from outside. She was a known case of hypertension and type 2 diabetes mellitus on treatment. She had attained menopause 7 years back. On abdominal examination tenderness was elicited in the left iliac fossa.

The patient was evaluated using ultrasonography (USG) and contrast-enhanced CT. On ultrasound evaluation, the left adnexa of the patient showed a well-defined cystic lesion measuring 8.5*6.9*6.5 cm with multiple fine internal echoes and chunky calcifications along its dependent part measuring 17*8 mm. Mild free fluid was noted in the left iliac fossa. No definite internal or peripheral vascularity could be elicited.

**Fig. 1:**
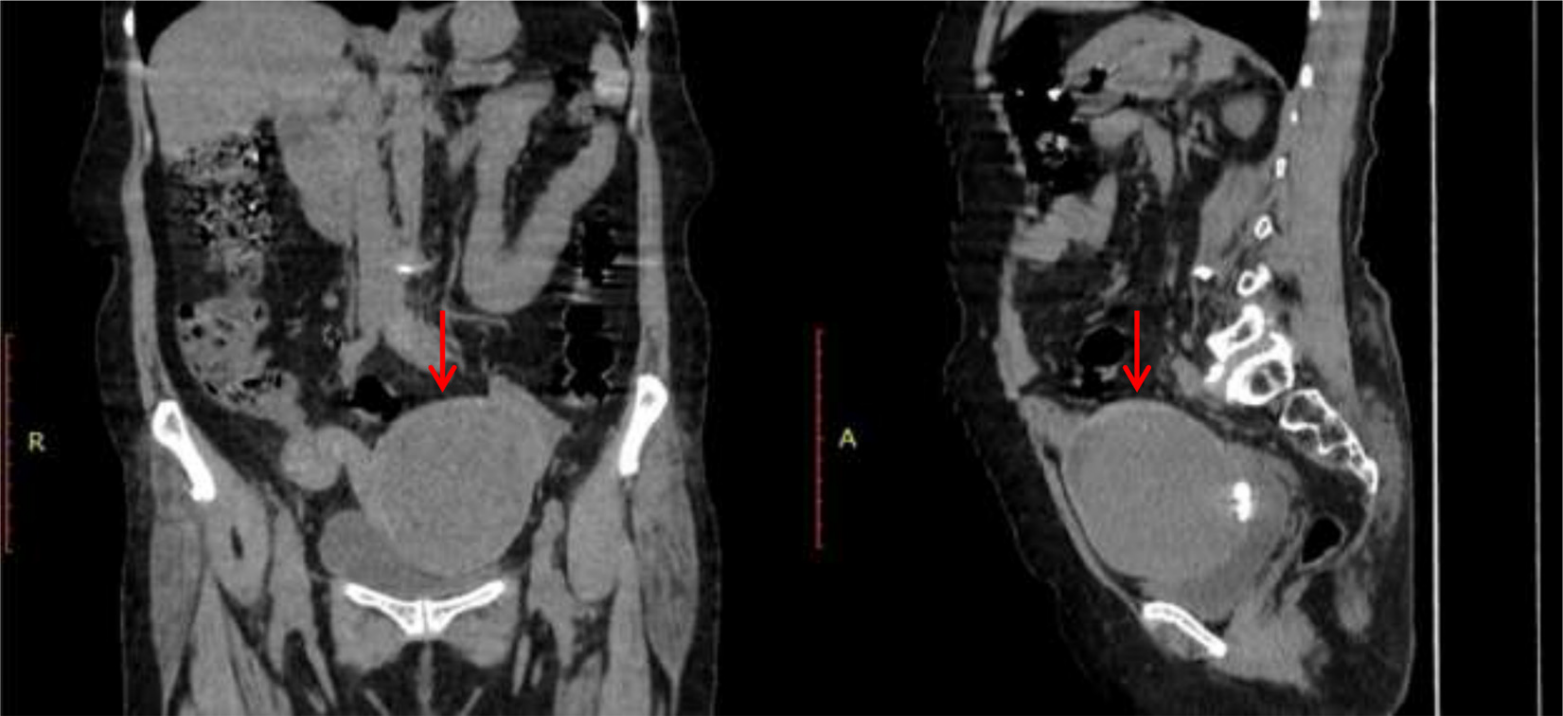
A 57-year-old who came with lower abdominal pain for 1 day. Coronal and sagittal sections show a large well defined abdominopelvic cystic thick walled lesion towards left side (red arrow).

**Fig. 2:**
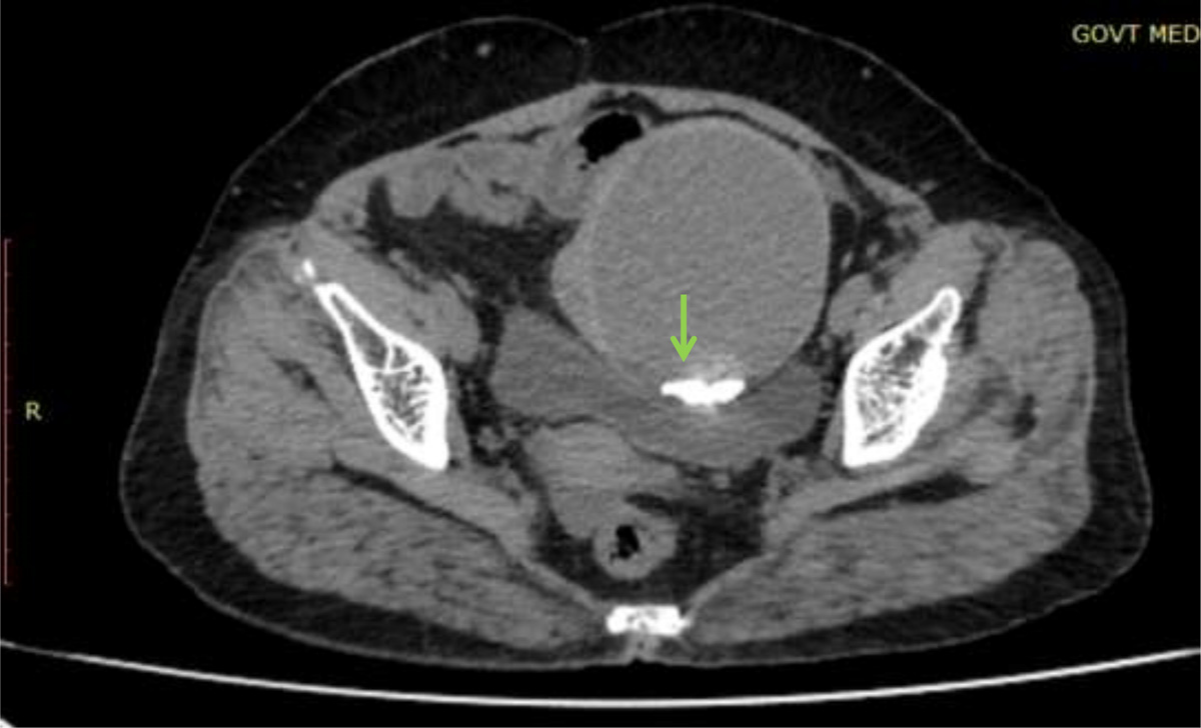
Axial CT images show chunky calcifications in the posterior aspect of the lesion. No septations or solid areas noted within the lesion (green arrow).

**Fig. 3:**
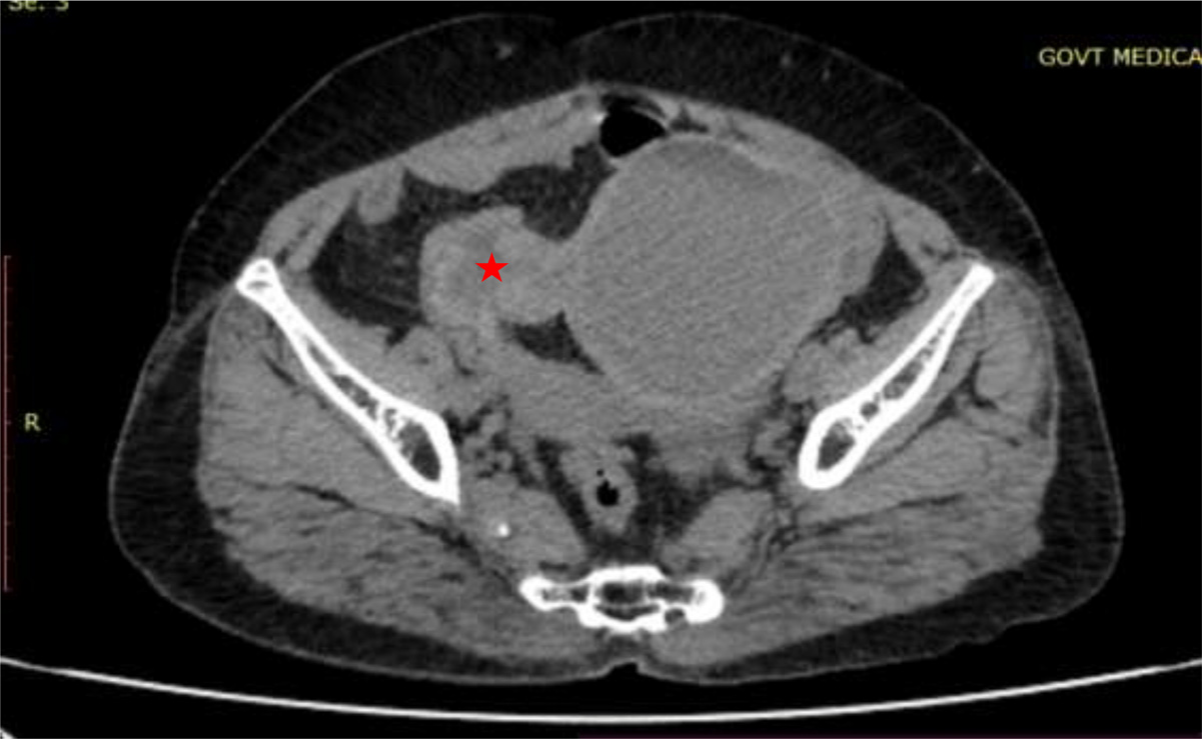
Axial CT image shows the pedicle of the lesion appearing thickened, edematous and twisted (red star).

**Fig. 4:**
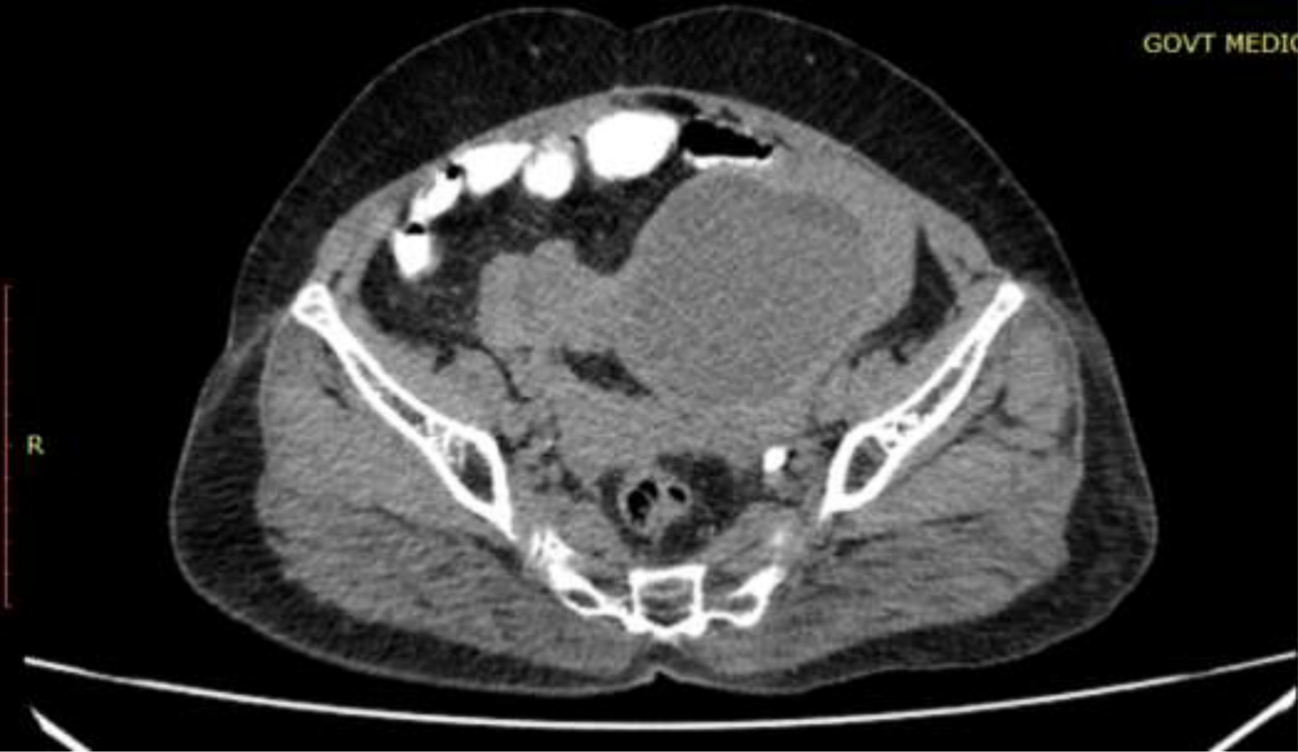
Postcontrast axial CT image shows no significant contrast enhancement of the wall of the lesion as well as the pedicle.

The patient was then assessed by contrast-enhanced CT study. CT images revealed a well-defined large abdominopelvic cystic lesion measuring 9* 7* 7.7 cm noted towards the left side of an atrophic uterus seemingly arising from the right ovary (Fig. 1). The right ovary could not be separately visualized. The lesion had thick enhancing walls, with a maximum thickness of 6 mm. Chunky calcifications, the largest measuring 18 mm, were seen in the posterior aspect of the lesion (Fig. 2). There were no septations or solid areas within the lesion. The pedicle of the lesion appeared thickened, edematous, and twisted (Fig. 3). Following contrast administration, the walls of the lesion, as well as the pedicle, did not show contrast enhancement (Fig. 4). A few ileal loops were seen closely abutting the lesion. However, there was no direct infiltration of adjacent structures. The lesion was seen to abut the dome of the bladder inferiorly. Mild free fluid was noted in pouch of Douglas. The absence of visible fat within the lesion hindered a confident diagnosis of ovarian teratoma.

**Fig. 5:**
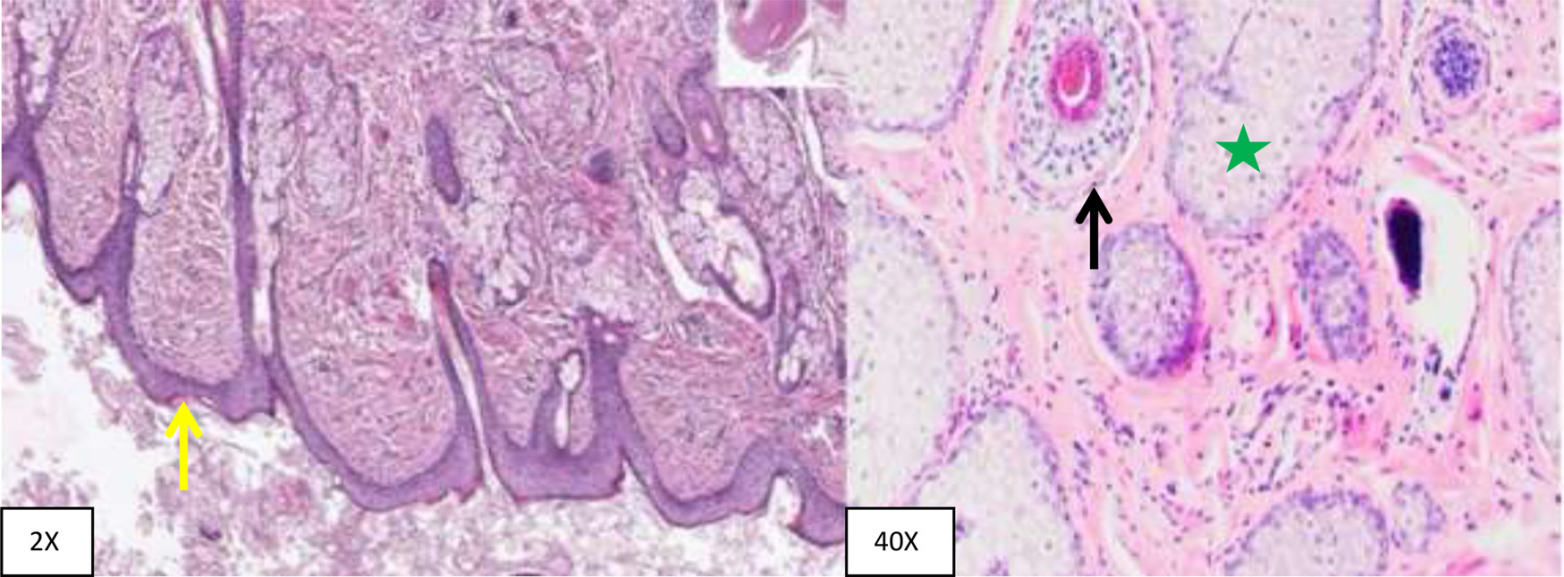
Histopathology specimens (At 2X and 40X original magnification using hematoxylin-eosin stain) show skin with keratinized squamous epithelium (yellow arrow), hair follicle (black arrow) and sebaceous gland (green star).

The patient underwent a total abdominal hysterectomy, bilateral salpingo-oophorectomy, and omental biopsy where a right ovarian cystic mass of size 8*8*7 cm was obtained which was twisted 2 times around the pedicle. The left ovary and uterus were found to be normal. Histopathological examination showed a mature ovarian cystic teratoma of 9*9*7 cm in size with evidence of torsion (Fig. 5).

## Discussion

Mature cystic teratomas (MCT) are germ cell tumors believed to arise from the primordial germ cell. Around 20%-25% of ovarian neoplasms are contributed by ovarian germ cell tumors [2,3]. Bilaterality is seen in 10%-17% of patients with majority occurring in women of childbearing age [4]. Various studies have reported the incidence of MCT to be about 1.2-14.2 cases per 100,000 per year [5], [6], [7]. Most patients are asymptomatic but can develop pain and sensation of abdominal fullness due to the mass or can become symptomatic when associated with complications like torsion [8].

Ovarian mature teratomas have a wide variety of imaging features. A good knowledge of radiological presentations of teratomas can help arrive at an accurate diagnosis. Though ultrasonography is often the first used imaging modality for the assessment of pelvic genital organs, the diagnosis of MCT may be difficult by the sole use of USG as they can have nonspecific appearance. However, a thorough search for characteristic signs such as rokitansky nodule, diffuse or regional high amplitude echoes, tip of the iceberg sign, dot–dash sign, fat fluid level or fluid-fluid level, floating balls sign and comet tail appearance should be undertaken [9]. CT has excellent sensitivity in detecting MCT. Typical findings include intralesional fat (incidence of 93%), tooth or calcifications (incidence of 56%), rokitansky nodule (incidence of 81%), and fat fluid level (incidence of 12%). Fat density numbers in MCTs range from -144 to -20 HU [10]. According to studies conducted by JN Buy and Eric K Outwater, the presence of fat within an ovarian cystic mass is diagnostic and is the most specific indicator of MCT [11,12].

The case presented here proposed a diagnostic dilemma since no evidence of intracystic fat could be demonstrated. Yamashita et al reported 12 cases of MCT without fat in the cystic component [13]. Sometimes small amounts of fat may be detected in the wall. Hence in patients showing the absence of intracystic fat, careful examination of cyst wall should be done to look for presence of fat [10,14]. Other atypical imaging manifestations include a pure fatty component in the cyst and, combination and collision tumors [15,16].

CT is better than MRI to visualize small fatty areas (mean diameter about 1 mm) which can be explained by a better signal-to-noise ratio in CT, thinner slices in CT, and magnetic field inhomogeneities in the boundary of the calcified structures [17]. MRI features of MCT include intratumoral fat, chemical shift artifact, and fat fluid level, rokitansky nodule, tuft of hair, palm tree-like protrusion, floating balls sign, and intratumoral keratinoid material [9].

Torsion and malignant degeneration constitute the 2 major complications of MCTs. Other complications which occur less frequently are tumor rupture and ovarian vein thrombophlebitis. Findings suggestive of torsion include deviation of the uterus to the twisted side, engorged blood vessels on the twisted side, engorged blood vessels on the twisted side, a mass with high sign intensity rim on T1- weighted MR images, a low signal intensity torsion knot, and thick, straight blood vessels that drape around the mass and cause complete absence of enhancement [18]. Malignant transformation occurs in the 6th or 7th decade and the most common type of malignant degeneration is squamous cell carcinoma which arises from the squamous lining of cyst [10].

There are many challenges faced by gynecologists in deciding upon the best surgical management for ovarian MCTs with the laparoscopic approach being considered as the gold standard for the management. The standard operation is oophorectomy except in younger women with a single small cyst [19].

## Patient consent

Written informed patient consent for publication has been obtained
